# Comparative outcomes of transcranial craniotomy and endoscopic endonasal surgery for craniopharyngioma: a single-center retrospective cohort study

**DOI:** 10.3389/fonc.2026.1845822

**Published:** 2026-06-24

**Authors:** Lei Zhang, Ben Li Li, Shuo Wei, Fa An Miao, Hong Fu Chen, Yue Chao Fan, Pei Zhi Ji, Hui Zhang

**Affiliations:** 1 Department of Neurosurgery, Affiliated Hospital of Xuzhou Medical University, Xuzhou, Jiangsu, China; 2 Department of Neurobiology, Xuzhou Medical University, Xuzhou, Jiangsu, China

**Keywords:** craniopharyngioma, endocrine outcome, endoscopic endonasal approach, gross total resection, retrospective cohort, transcranial craniotomy, visual outcome

## Abstract

**Background:**

The optimal surgical corridor for craniopharyngioma remains debated. Endoscopic endonasal approach (EEA) offers a direct midline trajectory to the sellar/suprasellar region, whereas transcranial approaches remain important for lesions with complex extension. We compared perioperative and follow-up outcomes between EEA and transcranial craniotomy in a single-center cohort, emphasizing detailed preoperative anatomical classification and domain-specific functional outcomes.

**Methods:**

We performed a retrospective cohort study of consecutive patients undergoing primary craniopharyngioma resection at the Affiliated Hospital of Xuzhou Medical University (January 2019–December 2024). Patients were grouped by surgical corridor used in routine care: transcranial craniotomy (TCA, n=33) or EEA (n=34). Preoperative MRI was reviewed for topography, cystic proportion, calcification, lateral extension beyond the internal carotid artery (ICA), vascular encasement, QST type, Kassam type, and Puget grade. Gross total resection (GTR) was defined as no residual enhancing tumor on MRI within 72 hours. Outcomes included extent of resection, length of stay, complications, axis-specific endocrine outcomes and replacement burden at 6 months, objective visual outcomes at 3 months, postoperative radiotherapy, and recurrence/progression at last follow-up.

**Results:**

Baseline demographic, anatomical, and endocrine characteristics were comparable between groups. EEA achieved a higher GTR rate (82.4% vs. 45.5%, p=0.002) and shorter hospital stay (16.19 ± 3.97 vs. 18.76 ± 4.23 days, p=0.019). At 6 months, headache/ICP-related symptoms improved in 90.9% of EEA patients versus 76.5% of TCA patients (p=0.047). Objective postoperative 3−month best-corrected visual acuity (BCVA, logMAR) was better after EEA (0.30 [0.12, 0.45] vs. 0.42 [0.22, 0.62], p=0.041). EEA was associated with lower rates of electrolyte imbalance (17.6% vs. 42.4%, p=0.021), transient diabetes insipidus (DI) (20.6% vs. 45.5%, p=0.028), persistent DI (≥3 months) (14.7% vs. 30.3%, p=0.048), and new hypopituitarism (any axis) (41.2% vs. 69.7%, p=0.022). The pituitary stalk was anatomically preserved more frequently in EEA cases (67.6% vs. 48.5%, p=0.042). Median follow-up was 35–37 months; recurrence/progression at last follow-up occurred in 8.8% of EEA patients versus 21.2% of TCA patients (p=0.047).

**Conclusion:**

In this retrospective cohort with detailed anatomical reclassification, EEA was associated with higher GTR, shorter hospitalization, and fewer short-term endocrine/electrolyte complications, with comparable overall safety. Because corridor selection is anatomy-driven and this unadjusted observational comparison is susceptible to confounding by indication, these findings should be interpreted as comparative outcomes among selected patients rather than causal evidence of superiority.

## 1 Introduction

Craniopharyngiomas are benign epithelial tumors arising from remnants of the craniopharyngeal duct epithelium and account for a small proportion of intracranial neoplasms, yet they cause disproportionate morbidity because of their proximity to the optic apparatus, pituitary stalk, and hypothalamus (1–3). Surgical resection remains the cornerstone of management, but the operative goal is constrained by adherence to or invasion of critical neurovascular structures that govern vision and hypothalamic-pituitary function (4–6).

Open transcranial approaches provide broad working angles and remain essential for tumors with marked lateral extension, dominant intraventricular growth, or complex vascular relationships. However, they may require brain and optic pathway manipulation, potentially increasing the risk of neurological and hypothalamic injury (4, 7). The endoscopic endonasal approach (EEA) offers a direct midline corridor with panoramic visualization and has expanded the operative reach to suprasellar and retrochiasmatic regions as skull base reconstruction matured, particularly with the vascularized nasoseptal flap technique (8–10).

Comparative evidence has suggested that EEA may improve extent of resection and visual outcomes in selected patients, while endocrine and hypothalamic morbidity remain major determinants of long-term quality of life (11–19). Importantly, corridor selection is anatomy-driven; therefore, retrospective comparisons are vulnerable to confounding by indication and require transparent anatomical characterization and cautious interpretation (11, 20, 21).

In this single-center retrospective cohort (2019–2024), we compared perioperative, functional, and tumor-control outcomes between transcranial craniotomy and EEA for primary craniopharyngioma resection. In response to limitations of traditional classifications, we reclassified tumors using the QST and Kassam systems and quantified hypothalamic involvement using Puget grade, alongside vascular and cystic features, to contextualize outcomes and support differentiated discussion (4, 11).

## 2 Data and methods

### 2.1 Study design and ethics

This study was designed as a single-center retrospective cohort study and is reported in accordance with the Strengthening the Reporting of Observational Studies in Epidemiology (STROBE) guidelines (22). The study protocol was approved by the institutional ethics committee of the Affiliated Hospital of Xuzhou Medical University (approval no. XYFY2025-KL421-01).

We screened consecutive patients with pathologically confirmed craniopharyngioma who underwent primary surgical resection between January 1, 2019 and December 31, 2024. Patients were grouped according to the surgical corridor performed during routine clinical care: transcranial approach (TCA, n=33) or endoscopic endonasal approach (EEA, n=34).

### 2.2 Inclusion and exclusion criteria

Inclusion criteria were: (1) histopathological confirmation of craniopharyngioma; (2) primary resection via a transcranial craniotomy or pure EEA; (3) availability of complete preoperative imaging and operative records; and (4) clinical and radiological follow-up of at least 6 months.

Exclusion criteria were: (1) prior surgery or radiotherapy for craniopharyngioma before the index operation at our center; (2) combined or staged endoscopic–transcranial approaches; (3) incomplete medical records or immediate loss to follow-up; and (4) other concurrent intracranial malignancies or systemic diseases expected to independently confound perioperative outcomes.

### 2.3 Preoperative imaging evaluation, tumor measurement, and classification

Preoperative contrast-enhanced MRI was reviewed to characterize tumor anatomy. Tumor maximum diameter was defined as the greatest dimension measured on any plane. Tumor volume was estimated using the ellipsoid approximation (A×B×C×π/6), where A, B, and C are the maximal orthogonal diameters on MRI. When a distinct cystic component was present, cystic volume was estimated similarly and cystic proportion was calculated as (cystic volume/total volume)×100%. Calcification was assessed using preoperative CT and recorded as present/absent.

Anatomical features relevant to corridor selection were recorded, including topography (intrasellar, intrasellar–suprasellar, suprasellar/retrochiasmatic, and extension to the third ventricle), lateral extension beyond the internal carotid artery (ICA) on coronal MRI/CTA, and vascular encasement (ICA/A1/PCom/PCA). Hypothalamic involvement was graded using the Puget classification (grade 0: none; grade 1: displacement; grade 2: disruption/invasion) (4).

To contextualize approach feasibility, tumors were reclassified using the QST system (Q: subdiaphragmatic/intrasellar; S: stalk/subarachnoidal; T: pars tuberalis with upward extension near the third-ventricle floor) and the Kassam infundibulum-based system (type I: preinfundibular; type II: transinfundibular; type III: retro/postinfundibular; type IV: isolated third ventricle) as described in the literature (7, 11). Classification was performed by two reviewers (neurosurgery and neuroradiology) and disagreements were resolved by consensus.

### 2.4 Surgical approach selection and surgeon experience

The surgical corridor (TCA vs. EEA) was selected preoperatively after multidisciplinary skull-base discussion (neurosurgery, neuroradiology, endocrinology, and otolaryngology when applicable). The decision was anatomy-driven based on MRI/CTA findings, including topography, lateral ICA extension, hypothalamic involvement, and vascular relationships. Patient factors (age, comorbidities) and surgeon expertise were also considered.

All operations were performed by two senior skull-base neurosurgeons with >20 years of experience in skull-base surgery and an established endoscopic endonasal program prior to 2019. For EEA cases, a binostril, two-surgeon technique was used.

### 2.5 Surgical techniques

Transcranial approach: Patients underwent craniopharyngioma resection via a tailored transcranial corridor (pterional, subfrontal, interhemispheric, or other) based on tumor anatomy. Microsurgical resection was performed under general anesthesia with careful preservation of the optic apparatus, perforators, hypothalamus, pituitary stalk, and major vessels.

**Figure 1:**
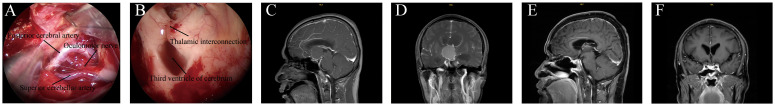
**(A)** A rectangular bone window (approximately 2.0 cm × 1.0 cm) created over the sella and planum. **(B)** Intraoperative view of the pituitary stalk. **(C, D)** Preoperative sagittal and coronal MRI. **(E, F)** Early postoperative sagittal and coronal MRI.

**Figure 2:**
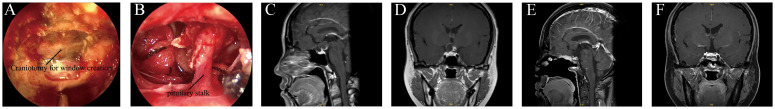
**(A)** Intraoperative view demonstrating the tumor and optic chiasm. **(B)** Rigid buttress/absorbable fixation material used for reconstruction. **(C, D)** Preoperative sagittal and coronal MRI. **(E, F)** Early postoperative sagittal and coronal MRI.

**Figure 3:**
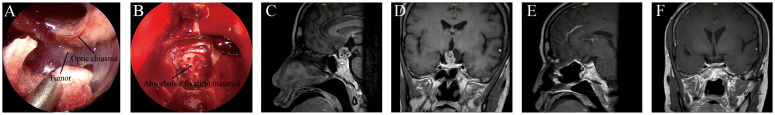
**(A)** Intraoperative view of the posterior cerebral artery. **(B)** Pedicled nasoseptal flap used for skull base reconstruction. **(C, D)** Preoperative sagittal and coronal MRI. **(E, F)** Early postoperative sagittal and coronal MRI.

**Figure 4:**
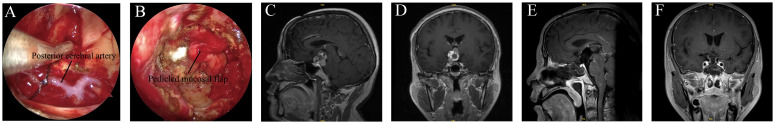
**(A)** Intraoperative view of the posterior cerebral artery, oculomotor nerve, and superior cerebellar artery. **(B)** Exposure of the third ventricle region. **(C, D)** Preoperative sagittal and coronal MRI. **(E, F)** Early postoperative sagittal and coronal MRI.

Endoscopic endonasal approach: Patients underwent an endoscopic endonasal transsphenoidal approach using a binostril technique. After harvesting a pedicled vascularized nasoseptal flap and creating a sellar/planum bone window, the tumor was debulked and dissected from surrounding neurovascular structures using a combination of sharp and blunt maneuvers under endoscopic visualization (**Figures 1**–**4**).

Skull base reconstruction and CSF leak management: Reconstruction was performed in a multilayer fashion using an inlay dural substitute, a rigid buttress, and coverage with the vascularized nasoseptal flap secured with fibrin glue (9). Lumbar drainage was not routinely used prophylactically; it was considered selectively for postoperative CSF rhinorrhea or high-risk leaks at the discretion of the treating team. Postoperative CSF rhinorrhea was managed with conservative measures and/or lumbar drainage, with endoscopic re-exploration when necessary.

### 2.6 Outcome definitions

Extent of resection was evaluated on early postoperative contrast-enhanced MRI performed within 72 hours and confirmed on subsequent follow-up MRI when available. Gross total resection (GTR) was defined as no residual enhancing tumor. Subtotal resection (STR) was defined as any visible residual tumor.

Visual outcomes: Preoperative and postoperative ophthalmologic assessments were reviewed in patients with documented preoperative visual impairment. Objective measures included best-corrected visual acuity (BCVA, logMAR) and visual field mean deviation (MD, dB) when available, evaluated preoperatively and at approximately 3 months postoperatively. BCVA and visual field outcomes were categorized as improved, stable, or worsened relative to baseline. New postoperative visual deterioration was recorded as any new or worsened visual deficit compared with baseline.

Symptom-domain outcomes: Headache/dizziness/intracranial pressure (ICP)-related symptoms and nausea/vomiting were assessed at 6 months, and improvement was defined as documented improvement or resolution among those symptomatic preoperatively.

Endocrine outcomes: Baseline endocrine status was recorded for all pituitary axes using endocrine notes, laboratory testing, and/or requirement for hormone replacement. New-onset hypopituitarism was defined as a new deficiency in at least one pituitary axis requiring replacement therapy at follow-up that was not present preoperatively. Diabetes insipidus (DI) was categorized as transient or persistent using a prespecified time threshold of 3 months (persistent DI ≥3 months). Axis-specific new deficits and replacement burden were assessed at 6 months. Anatomical pituitary stalk preservation was determined from operative records.

Tumor recurrence/progression was defined as radiographic evidence of new tumor after GTR or growth of residual tumor after STR on surveillance MRI. Follow-up duration was calculated from the date of surgery to the most recent clinical or radiographic follow-up.

### 2.7 Statistical analysis

SPSS version 26.0 was used for statistical analyses. Continuous variables are reported as mean ± standard deviation (SD) or median (interquartile range, IQR) as appropriate, and categorical variables as counts and percentages. Between-group comparisons were performed using Student’s t-test or Mann–Whitney U test for continuous variables and chi-square test or Fisher’s exact test for categorical variables. A two-sided p<0.05 was considered statistically significant.

Because this is an anatomy-driven retrospective comparison with a limited sample size and event counts for several outcomes, we did not perform propensity-score modeling or multivariable adjustment; results are presented as unadjusted comparative outcomes and interpreted with explicit consideration of potential confounding by indication.

## 3 Results

### 3.1 Baseline demographic, clinical, and endocrine characteristics

A total of 67 patients met inclusion criteria, including 33 undergoing TCA and 34 undergoing EEA. Baseline demographic and clinical characteristics were comparable between groups (**Table 1**). The mean age was 39.18 ± 19.85 years in the TCA group and 42.47 ± 15.95 years in the EEA group (p=0.457), and the sex distribution was similar (male: 45.5% vs. 44.1%, p=0.893). Tumor size and morphology were comparable, including maximum diameter, total volume, and cystic proportion (all p>0.05). Hydrocephalus was uncommon (one patient in each group).

**Table 1:** Baseline demographic, clinical, and endocrine characteristics.

Variable	TCA (n=33)	EEA (n=34)	p value
Age, years (mean ± SD)	39.18 ± 19.85	42.47 ± 15.95	0.457
Sex, male, n (%)	15 (45.5%)	15 (44.1%)	0.893
Max tumor diameter, cm (mean ± SD)	2.85 ± 0.31	2.91 ± 0.37	0.427
Tumor volume, cm³ (median [IQR])	12.25 [8.42, 14.88]	12.19 [8.15, 14.22]	0.620
Cystic proportion, % (mean ± SD)	59.95 ± 32.85	67.82 ± 28.40	0.300
Calcification, n (%)	29 (87.9%)	24 (70.6%)	0.086
Hydrocephalus, n (%)	1 (3.0%)	1 (2.9%)	1.000
Preoperative visual impairment, n (%)	25 (75.8%)	27 (79.4%)	0.782
Preoperative headache/dizziness/ICP symptoms, n (%)	17 (51.5%)	22 (64.7%)	0.275
Preoperative nausea/vomiting, n (%)	9 (27.3%)	11 (32.4%)	0.791
Pituitary dysfunction at baseline, n (%)			
Adrenal insufficiency	18 (54.5%)	23 (67.6%)	0.235
Central hypothyroidism	14 (42.4%)	19 (55.9%)	0.296
Central hypogonadism	19 (57.6%)	23 (67.6%)	0.307
GH deficiency	2 (6.1%)	3 (8.8%)	1.000
Hyperprolactinemia	6 (18.2%)	10 (29.4%)	0.271
Diabetes insipidus	5 (15.2%)	7 (20.6%)	0.754
No. of deficient pituitary axes (median [IQR])	2 [1, 2]	2 [1, 3]	0.316

Preoperative symptoms were frequent and balanced between groups, including visual impairment (75.8% vs. 79.4%), headache/ICP-related symptoms (51.5% vs. 64.7%), and nausea/vomiting (27.3% vs. 32.4%). Baseline endocrine dysfunction was common, with median 2 deficient pituitary axes in both groups (p=0.316), and no significant intergroup differences across axis-specific baseline deficits (**Table 1**).

### 3.2 Preoperative imaging anatomy and classification

Preoperative anatomical distributions were comparable between groups (**Table 2**). Tumor topography (intrasellar–suprasellar, suprasellar/retrochiasmatic, and third-ventricle extension) showed no significant intergroup difference (p=0.853). Lateral extension beyond the ICA (27.3% vs. 32.4%, p=0.615) and vascular encasement (24.2% vs. 29.4%, p=0.786) were also similar.

**Table 2:** Preoperative imaging anatomy and classification.

Variable	TCA (n=33)	EEA (n=34)	p value
Topography, n (%)			0.853
Intrasellar	0 (0.0%)	0 (0.0%)	
Intrasellar–suprasellar	11 (33.3%)	14 (41.2%)	
Suprasellar/retrochiasmatic	12 (36.4%)	11 (32.4%)	
Extension to third ventricle	10 (30.3%)	9 (26.5%)	
Lateral extension beyond ICA, n (%)	9 (27.3%)	11 (32.4%)	0.615
Vascular encasement (ICA/A1/PCom/PCA), n (%)	8 (24.2%)	10 (29.4%)	0.786
QST type, n (%)			0.865
Q type	12 (36.4%)	13 (38.2%)	
S type	11 (33.3%)	10 (29.4%)	
T type	10 (30.3%)	11 (32.4%)	
Kassam type, n (%)			0.792
Type I	1 (3.0%)	2 (5.9%)	
Type II	12 (36.4%)	13 (38.2%)	
Type III	11 (33.3%)	10 (29.4%)	
Type IV	9 (27.3%)	9 (26.5%)	
Puget grade, n (%)			0.887
Grade 0	3 (9.1%)	4 (11.8%)	
Grade 1	14 (42.4%)	15 (44.1%)	
Grade 2	16 (48.5%)	15 (44.1%)	

QST types-Q, subdiaphragmatic/intrasellar; S, stalk/subarachnoidal; T, parstuberalis with upward extension near third-ventricle floor. Kassam types-I, preinfundibular; II, transinfundibular; III, retro/postinfundibular; IV ,isolated third ventricle. Puget grade-0, none; 1, displacement; 2, disruption/invasion. ICA, internal carotid artery.

After reclassification, QST (Q/S/T) and Kassam (I–IV) distributions did not differ significantly between groups (p=0.865 and p=0.792, respectively). Hypothalamic involvement graded by Puget classification was also comparable (p=0.887).

### 3.3 Surgical outcomes, recovery, and complications

Among TCA cases, the most common corridor was pterional (66.7%), followed by subfrontal (21.2%) and interhemispheric/other approaches (12.1%) (**Table 3**). Operative time and estimated blood loss were similar between groups (p=0.218 and p=0.335, respectively).

**Table 3:** Surgical outcomes, recovery, complications, and follow-up.

Outcome	TCA (n=33)	EEA (n=34)	p value
Transcranial corridor distribution, n (%)			–
Pterional	22 (66.7%)	–	
Subfrontal	7 (21.2%)	–	
Interhemispheric/Other	4 (12.1%)	–	
Operative time, min (mean ± SD)	231.42 ± 27.29	239.74 ± 27.44	0.218
Estimated blood loss, mL (mean ± SD)	246.36 ± 118.87	219.12 ± 110.90	0.335
Gross total resection, n (%)	15 (45.5%)	28 (82.4%)	0.002
Length of hospital stay, days (mean ± SD)	18.76 ± 4.23	16.19 ± 3.97	0.019
Headache/ICP symptom improvement at 6 months, n/N (%)	13/17 (76.5%)	20/22 (90.9%)	0.047
Nausea/vomiting improvement at 6 months, n/N (%)	7/9 (77.8%)	9/11 (81.8%)	0.812
Postoperative hemorrhage, n (%)	2 (6.1%)	0	0.239
CSF rhinorrhea, n (%)	0	3 (8.8%)	0.114
Pseudoaneurysm, n (%)	0	1 (2.9%)	1.000
Cranial nerve injury, n (%)	1 (3.0%)	2 (5.9%)	1.000
Intracranial infection, n (%)	4 (12.1%)	2 (5.9%)	0.427
Mortality, n (%)	1 (3.0%)	1 (2.9%)	1.000
Electrolyte imbalance, n (%)	14 (42.4%)	6 (17.6%)	0.021
Diabetes insipidus (transient), n (%)	15 (45.5%)	7 (20.6%)	0.028
Diabetes insipidus (persistent ≥3 months), n (%)	10 (30.3%)	5 (14.7%)	0.048
New hypopituitarism (any axis), n (%)	23 (69.7%)	14 (41.2%)	0.022
Follow-up duration (months), median (range)	37 (6–71)	35 (6–70)	0.790
Postoperative radiotherapy, n (%)	6 (18.2%)	4 (11.8%)	0.450
Tumor recurrence/progression, n (%)	7 (21.2%)	3 (8.8%)	0.047

CSF, cerebrospinal fluid; DI, diabetes insipidus; ICP, intracranial pressure.

EEA achieved a significantly higher radiographic GTR rate compared with TCA (82.4% vs. 45.5%, p=0.002) and was associated with a shorter length of hospital stay (16.19 ± 3.97 vs. 18.76 ± 4.23 days, p=0.019). At 6 months, headache/ICP-related symptoms improved in 90.9% of symptomatic EEA patients compared with 76.5% in the TCA group (p=0.047), whereas nausea/vomiting improvement rates were comparable (81.8% vs. 77.8%, p=0.812).

Regarding complications, the EEA group had lower rates of electrolyte imbalance (17.6% vs. 42.4%, p=0.021), transient DI (20.6% vs. 45.5%, p=0.028), persistent DI (≥3 months) (14.7% vs. 30.3%, p=0.048), and new hypopituitarism (any axis) (41.2% vs. 69.7%, p=0.022). Rates of postoperative hemorrhage, cranial nerve injury, intracranial infection, and mortality did not differ significantly (all p>0.05). CSF rhinorrhea occurred in three EEA patients (8.8%) and pseudoaneurysm occurred in one EEA patient (2.9%).

### 3.4 Visual outcomes

Among patients with preoperative visual impairment (TCA: 25/33; EEA: 27/34), preoperative BCVA (logMAR) was similar between groups (median 0.50 vs. 0.52, p=0.683) (**Table 4**). At 3 months postoperatively, BCVA was better in the EEA group (median 0.30 vs. 0.42, p=0.041). The proportion of patients classified as having improved BCVA was numerically higher after EEA (51.9% vs. 28.0%), although this did not reach statistical significance (p=0.160). New postoperative visual deterioration was uncommon and comparable between groups (5.9% vs. 9.1%, p=0.672).

**Table 4:** Visual outcomes in patients with preoperative visual impairment.

Visual outcome	TCA	EEA	p value
Patients with preop visual impairment, n (%)	25 (75.8%)	27 (79.4%)	0.782
Preop BCVA (logMAR), median [IQR]	0.50 [0.30, 0.70]	0.52 [0.32, 0.72]	0.683
Postop 3-month BCVA (logMAR), median [IQR]	0.42 [0.22, 0.62]	0.30 [0.12, 0.45]	0.041
BCVA improved, n (%)	7 (28.0%)	14 (51.9%)	0.160
BCVA stable, n (%)	14 (56.0%)	11 (40.7%)	0.120
BCVA worsened, n (%)	4 (16.0%)	2 (7.4%)	0.400
Patients with preop visual field defect, n (%)	22 (66.7%)	24 (70.6%)	0.780
Preop visual field MD (dB), mean ± SD	-8.6 ± 2.4	-9.0 ± 2.5	0.526
Postop 3-month visual field MD (dB), mean ± SD	-6.3 ± 2.2	-5.4 ± 1.9	0.068
Visual field improved, n (%)	13 (59.1%)	19 (79.2%)	0.140
Visual field stable, n (%)	7 (31.8%)	4 (16.7%)	0.200
Visual field worsened, n (%)	2 (9.1%)	1 (4.2%)	0.600
New postoperative visual deterioration, n (%)	3 (9.1%)	2 (5.9%)	0.672

BCVA, best-corrected visual acuity (logMAR). Visual field MD, mean deviation (dB). Percentages for BCVA outcomes are calculated among patients with preoperative visual impairment; percentages for visual field outcomes are calculated among patients with preoperative visual field defect. New postoperative visual deterioration is reported among all patients.

Among patients with preoperative visual field defect (TCA: 22/33; EEA: 24/34), baseline visual field MD was similar (−8.6 ± 2.4 vs. −9.0 ± 2.5 dB, p=0.526). Postoperative 3−month MD showed a trend toward greater improvement after EEA (−5.4 ± 1.9 vs. −6.3 ± 2.2 dB, p=0.068), with numerically higher visual field improvement rates (79.2% vs. 59.1%, p=0.140) (**Table 4**).

### 3.5 Endocrine outcomes and replacement burden

Anatomical pituitary stalk preservation was documented more frequently in the EEA group (67.6% vs. 48.5%, p=0.042) (**Table 5**). Axis-specific new endocrine deficits at 6 months were numerically lower in the EEA group across axes, although differences were not statistically significant for individual axes (all p>0.05). Persistent DI at 6 months was significantly lower after EEA (14.7% vs. 30.3%, p=0.048). The median number of axes requiring replacement at 6 months was lower in the EEA group (1 [1,2] vs. 2 [1,3]), with a trend toward significance (p=0.062) (**Table 5**).

**Table 5:** Axis-specific endocrine outcomes and replacement burden.

Endocrine outcome	TCA (n=33)	EEA (n=34)	p value
Stalk preserved (anatomically), n (%)	16 (48.5%)	23 (67.6%)	0.042
New adrenal insufficiency, n (%)	7 (21.2%)	3 (8.8%)	0.175
New central hypothyroidism, n (%)	8 (24.2%)	4 (11.8%)	0.213
New central hypogonadism, n (%)	9 (27.3%)	5 (14.7%)	0.192
New GH deficiency, n (%)	12 (36.4%)	7 (20.6%)	0.138
Persistent DI, n (%)	10 (30.3%)	5 (14.7%)	0.048
No. of axes requiring replacement, median [IQR]	2 [1, 3]	1 [1, 2]	0.062

All endocrine outcomes were assessed at 6 months postoperatively. New deficits indicate new hormone replacement requirements not present before surgery. Replacement burden is defined as the number of pituitary axes requiring ongoing hormone replacement therapy. DI, diabetes insipidus; IQR, interquartile range.

### 3.6 Follow-up and tumor control

Median follow-up duration was similar between groups (TCA: 37 months [range 6–71] vs. EEA: 35 months [range 6–70], p=0.790) (**Table 3**). Postoperative radiotherapy was administered to 18.2% of TCA patients and 11.8% of EEA patients (p=0.450), primarily for residual disease after STR. At last follow-up, tumor recurrence/progression was observed in 7 TCA patients (21.2%) and 3 EEA patients (8.8%) (p=0.047).

## 4 Discussion

In this retrospective cohort of 67 consecutive patients undergoing primary craniopharyngioma resection, EEA was associated with a higher radiographic GTR rate and shorter hospitalization compared with transcranial craniotomy. We strengthened anatomical characterization by reclassifying tumors using QST and Kassam systems and grading hypothalamic involvement by Puget classification, alongside vascular and cystic features. The distributions of these key anatomical variables were similar between groups, which supports comparability of the cohorts at baseline; nevertheless, corridor selection was individualized and anatomy-driven, and unmeasured confounding remains possible.

Extent of resection remains a key surrogate for tumor control in craniopharyngioma surgery. The higher GTR rate observed after EEA in our cohort (82.4%) is consistent with comparative series and meta-analyses reporting improved resection rates for selected midline tumors through an endonasal corridor (11–14, 20, 23, 24). The endoscope provides close-up panoramic visualization and multi-angled inspection of the tumor-stalk-hypothalamus interface, potentially facilitating meticulous sharp dissection and early identification of residual tumor in retrochiasmatic spaces (8, 25). However, GTR is not a standalone quality metric; in some cases, an intended subtotal resection may be chosen to preserve hypothalamic integrity when dense adherence is encountered. Because operative intent was not prospectively recorded in all cases, we could not reliably distinguish planned STR from incomplete resection, which should be considered when interpreting GTR differences.

Vision is a major determinant of patient-centered outcome. In our cohort, objective postoperative BCVA at 3 months was better after EEA, and visual field indices showed a favorable trend. These findings align with the theoretical advantage of EEA’s direct midline trajectory to the optic chiasm without optic nerve retraction, which has been associated with improved visual outcomes in prior comparative analyses (7, 11–13, 15, 20, 23). Although categorical improvement rates in our dataset did not reach statistical significance, the objective BCVA difference supports clinically relevant visual recovery among selected EEA patients.

Endocrine morbidity remains a central trade-off in craniopharyngioma surgery and is strongly influenced by baseline dysfunction, hypothalamic involvement, and pituitary stalk management (4–6, 18, 26, 27). By adding detailed baseline endocrine profiling and axis-specific new deficits at 6 months, we aimed to improve interpretability of endocrine comparisons. EEA was associated with lower rates of persistent DI and overall new hypopituitarism and with a higher stalk preservation rate, consistent with the concept that the endonasal corridor can facilitate stalk identification and preservation in appropriate cases (5, 26). Nevertheless, endocrine outcomes are vulnerable to confounding by tumor-stalk anatomy and surgical decision-making; long-term endocrine burden and hypothalamic morbidity require standardized prospective assessment.

Skull base reconstruction is critical for minimizing CSF leak after EEA. We used multilayer reconstruction with an inlay graft, rigid buttress, and vascularized nasoseptal flap, a technique associated with reduced postoperative leak rates in expanded endonasal surgery (9, 28). In our cohort, postoperative CSF rhinorrhea occurred in 8.8% of EEA patients and was managed according to institutional practice. One pseudoaneurysm occurred after EEA; although rare, this complication highlights the need for meticulous vascular handling and vigilant postoperative surveillance, particularly when working near the ICA and perforators.

Long-term tumor control is the ultimate endpoint. Unlike our initial draft emphasizing a 6−month minimum, we now report recurrence/progression at last follow-up with a median follow-up of approximately 3 years. Recurrence/progression was lower in the EEA group; however, this composite endpoint incorporates both recurrence after GTR and progression after STR and may be influenced by extent of resection, radiotherapy utilization, and follow-up intensity.

This study has limitations inherent to its retrospective, single-center design. First, approach selection was anatomy-driven; although key anatomical variables (QST, Kassam, Puget grade, ICA lateral extension, and vascular encasement) were comparable between groups, confounding by indication cannot be excluded. Second, because of the limited sample size and event counts for several outcomes, we did not perform propensity-score modeling or multivariable adjustment, and results should not be interpreted as causal evidence of superiority. Third, hypothalamic outcomes (e.g., standardized measures of hypothalamic obesity and quality of life) were not systematically captured, despite their clinical importance (4, 15, 18). Finally, visual and endocrine follow-up assessments were based on routine clinical practice, and longer standardized follow-up is needed to fully characterize durable functional outcomes.

Despite these limitations, our study provides a contemporary single-center comparison with detailed anatomical reclassification, baseline endocrine profiling, objective visual metrics, and long-term follow-up reporting. These data may help inform corridor selection discussions and highlight endpoints that should be standardized in future multicenter prospective studies.

## 5 Conclusion

In a single-center retrospective cohort with detailed anatomical reclassification, EEA was associated with higher radiographic GTR, shorter hospitalization, better objective postoperative BCVA, and fewer endocrine/electrolyte complications compared with transcranial craniotomy among selected patients. Given anatomy-driven corridor selection and the unadjusted observational design, these findings should be interpreted as comparative outcomes rather than causal proof of superiority. Prospective studies with standardized hypothalamic, endocrine, visual, and quality-of-life endpoints are warranted.

## Data Availability

The raw data supporting the conclusions of this article will be made available by the authors, without undue reservation.
