# Supplementary material for: Comparative outcomes of transcranial craniotomy and endoscopic endonasal surgery for craniopharyngioma: a single-center retrospective cohort study
Source: Front Oncol. 2026 Jun 24;16:1845822. doi: 10.3389/fonc.2026.1845822 (PMC13341450; doi:10.3389/fonc.2026.1845822)
Supplement: Supplementary file 1 [file DataSheet1.docx]

**STROBE Checklist for "Comparative Outcomes of Transcranial Craniotomy and Endoscopic Endonasal Surgery for Craniopharyngioma: A Single-Center Retrospective Cohort Study"**

**Title and Abstract**

1. (a) Indicate the study’s design with a commonly used term in the title or the abstract.

Answer: This study is explicitly identified as a retrospective cohort study in both the title and the abstract.

(b) Provide in the abstract an informative and balanced summary of what was done and what was found.

Answer:

Background: The optimal surgical corridor for craniopharyngioma remains debated. Endoscopic endonasal approach (EEA) offers a direct midline trajectory to the sellar/suprasellar region, whereas transcranial approaches remain important for lesions with complex extension. We compared perioperative and follow-up outcomes between EEA and transcranial craniotomy in a single-center cohort, emphasizing detailed preoperative anatomical classification and domain-specific functional outcomes.

Methods: We performed a retrospective cohort study of consecutive patients undergoing primary craniopharyngioma resection at the Affiliated Hospital of Xuzhou Medical University (January 2019–December 2024). Patients were grouped by surgical corridor used in routine care: transcranial craniotomy (TCA, n=33) or EEA (n=34). Preoperative MRI was reviewed for topography, cystic proportion, calcification, lateral extension beyond the internal carotid artery (ICA), vascular encasement, QST type, Kassam type, and Puget grade. Gross total resection (GTR) was defined as no residual enhancing tumor on MRI within 72 hours. Outcomes included extent of resection, length of stay, complications, axis-specific endocrine outcomes and replacement burden at 6 months, objective visual outcomes at 3 months, postoperative radiotherapy, and recurrence/progression at last follow-up.

Results: Baseline demographic, anatomical, and endocrine characteristics were comparable between groups. EEA achieved a higher GTR rate (82.4% vs. 45.5%, p=0.002) and shorter hospital stay (16.19±3.97 vs. 18.76±4.23 days, p=0.019). At 6 months, headache/ICP-related symptoms improved in 90.9% of EEA patients versus 76.5% of TCA patients (p=0.047). Objective postoperative 3‑month best-corrected visual acuity (BCVA, logMAR) was better after EEA (0.30 [0.12, 0.45] vs. 0.42 [0.22, 0.62], p=0.041). EEA was associated with lower rates of electrolyte imbalance (17.6% vs. 42.4%, p=0.021), transient diabetes insipidus (DI) (20.6% vs. 45.5%, p=0.028), persistent DI (≥3 months) (14.7% vs. 30.3%, p=0.048), and new hypopituitarism (any axis) (41.2% vs. 69.7%, p=0.022). The pituitary stalk was anatomically preserved more frequently in EEA cases (67.6% vs. 48.5%, p=0.042). Median follow-up was 35–37 months; recurrence/progression at last follow-up occurred in 8.8% of EEA patients versus 21.2% of TCA patients (p=0.047).

Conclusion: In this retrospective cohort with detailed anatomical reclassification, EEA was associated with higher GTR, shorter hospitalization, and fewer short-term endocrine/electrolyte complications, with comparable overall safety. Because corridor selection is anatomy-driven and this unadjusted observational comparison is susceptible to confounding by indication, these findings should be interpreted as comparative outcomes among selected patients rather than causal evidence of superiority.

Keywords: Craniopharyngioma; Transcranial craniotomy; Endoscopic endonasal approach; Gross total resection; Visual outcome; Endocrine outcome; Retrospective cohort

2. Background/Rationale: Explain the scientific background and rationale for the investigation being reported.

Answer: Craniopharyngiomas are histologically benign but clinically challenging tumors due to their critical adjacency to the optic apparatus and hypothalamus. While open transcranial craniotomy (TCC) provides wide operative exposure, the endoscopic endonasal approach (EEA) has increasingly been adopted for its direct ventral trajectory. However, systematic comparisons regarding key surgical, objective visual, and long-term endocrine outcomes remain debated. Existing studies are frequently limited by inconsistent classification systems, subjective outcome reporting, or failure to evaluate specific anatomical complexities (e.g., ICA extension, Puget grading). The scientific rationale for this investigation is to provide a detailed comparative evaluation using standardized anatomical grading and objective functional endpoints (e.g., BCVA, VF MD, 6-month endocrine burden) to better inform surgical decision-making.

3. Objectives: State specific objectives, including any prespecified hypotheses.

Answer: The objective of this study was to compare the unadjusted clinical outcomes of EEA versus TCC for craniopharyngioma across three domains: (1) surgical efficacy (GTR rates, operative parameters); (2) functional recovery (objective visual acuity/field changes and new-onset endocrine deficits at 6 months); and (3) safety profiles (30-day complication rates). We hypothesized that EEA would be associated with favorable visual and endocrine outcomes in appropriately selected patients.

**Methods**

4. Study Design: Present key elements of study design early in the paper.

Answer: This was a retrospective, single-center, comparative cohort study. We analyzed consecutive patients with craniopharyngioma who underwent primary surgical resection between January 1, 2019, and December 31, 2024. Patients were categorized into two unadjusted cohorts based on the executed surgical approach: TCC (n=33) or EEA (n=34).

5. Setting: Describe the setting, locations, and relevant dates, including periods of recruitment, exposure, follow-up, and data collection.

Answer: The study was conducted at the Department of Neurosurgery, Affiliated Hospital of Xuzhou Medical University. The enrollment period spanned from January 2019 to December 2024. Exposure was the surgical intervention. Follow-up data were collected from the immediate postoperative period through the most recent outpatient clinical encounter (with the latest follow-up updated to early 2025).

6. Participants: Give the eligibility criteria, and the sources and methods of selection of participants. Describe methods of follow-up.

Answer: Eligibility Criteria: Consecutive patients who underwent primary surgical resection for craniopharyngioma. Exclusions included prior radiation/surgery for craniopharyngioma, predominantly lateral/intraventricular tumors clearly unsuited for the alternative approach, and patients lost to follow-up within the first postoperative month.
Selection: Identified via the hospital EMR system. Patients were assigned to the TCC or EEA group based on the actual procedure performed.
Follow-up: Extracted from clinical records, including routine neuroimaging, ophthalmologic examinations, and endocrinology evaluations at 3-6 months and latest clinical encounters.

7. Variables: Clearly define all outcomes, exposures, predictors, potential confounders, and effect modifiers. Give diagnostic criteria, if applicable.

Answer:

Exposure: Surgical approach (TCC vs. EEA).

Outcomes:

Primary Outcome: GTR, defined as the absence of residual enhancing tumor on 72-hour postoperative MRI.

Secondary Outcomes:

Objective Visual Outcomes: Changes in Best-Corrected Visual Acuity (BCVA) via Snellen charts and Visual Field Mean Deviation (VF MD) via Humphrey perimetry at 6 months.

Endocrine Outcomes: Pituitary stalk preservation rate, 30-day transient/permanent diabetes insipidus (DI), and new-onset specific axis deficits at 6 months.

Perioperative Parameters: Operative time, blood loss, hospital stay, and complication rates (CSF leak, hemorrhage, infection, electrolyte imbalance).

Covariates (Anatomical Classifications): Preoperative MRI was evaluated using QST classification, Kassam classification, Puget hypothalamic grading, degree of cystic/solid components, and lateral extension beyond the supraclinoid ICA.

8. Data Sources/Measurement: For each variable of interest, give sources of data and details of methods of assessment (measurement). Describe comparability of assessment methods if there is more than one group.

Answer: Data were extracted from the EMR and PACS.

For each variable, the source and measurement method were as follows:

Exposure (Surgical Approach): Categorically determined from the primary procedural description in the operative report.

Objective visual function was quantified using Snellen charts (BCVA) and Humphrey perimetry (MD).

Endocrine status was determined by laboratory hormone panels and the requirement for replacement therapy evaluated by an endocrinologist.

Gross Total Resection (GTR): Assessed via postoperative MRI (performed within 72 hours) in the PACS. Measurement was based on the official neuroradiology report, which utilized a standardized criterion: the absence of any nodular or solid enhancing residual tumor on T1-weighted gadolinium-enhanced sequences.

Operative Parameters: Operative time (skin incision to closure) and estimated blood loss were obtained directly from the anesthesia record.

Postoperative Recovery: Length of hospital stay was calculated from the date of surgery to the date of discharge, recorded in the discharge summary. Symptomatic improvement was determined by comparing documented symptoms in the preoperative clinical notes with those recorded in the first follow-up outpatient clinic note (1-3 months postoperatively).

Complications: Diagnoses were extracted from progress notes, discharge summaries, and laboratory reports. Specific diagnostic criteria were applied: Diabetes insipidus required clinical documentation plus supportive serum/urine osmolality; Hypopituitarism required an endocrinologist’s diagnosis and initiation of replacement therapy; Electrolyte imbalance was defined as a value beyond the normal range requiring intervention; Cranial nerve injury required documentation on neurological examination; Surgical complications (hemorrhage, infection) required radiographic or microbiological confirmation noted in relevant reports.

Comparability of Assessment: All data sources and measurement methods were identical for both the open craniotomy and endoscopic resection groups. All patients underwent the same postoperative imaging protocol (MRI within 72 hours), and all clinical documentation followed the same departmental templates and standards. This ensured that the assessment of exposures, outcomes, and confounders was comparable across groups.

9. Bias: Describe any efforts to address potential sources of bias.

Answer:Due to the retrospective design and the lack of propensity score matching, confounding by indication (selection bias) is the primary inherent bias, as surgical approach was determined by tumor anatomy and surgeon preference rather than randomization. To address this, we robustly categorized and reported tumor anatomy using four standardized systems (QST, Kassam, Puget, ICA extension) to demonstrate the baseline anatomical comparability of the two cohorts. Furthermore, we explicitly refrained from causal assertions, framing the study strictly as a descriptive comparative analysis of associated outcomes. Measurement bias was minimized by using objective endpoints (e.g., BCVA, VF MD, radiologist-read GTR) rather than subjective patient-reported symptoms.

10. Study Size: Explain how the study size was arrived at.

Answer: The sample size was determined by the consecutive availability of eligible primary craniopharyngioma cases treated at our institution during the predefined 6-year period (2019-2024), resulting in a final cohort of 67 patients. No formal prospective sample size calculation was performed.

11. Quantitative Variables: Explain how quantitative variables were handled in the analyses. If applicable, describe which groupings were chosen and why.

Answer: Continuous variables were checked for normality. Normally distributed variables (e.g., hospital stay) were presented as mean ± SD and compared using independent t-tests. Non-normally distributed variables (e.g., tumor volume, follow-up months) were presented as median (range or IQR) and compared using the Mann-Whitney U test. Categorical variables were presented as frequencies/percentages.

1. Statistical Methods:

(a) Describe all statistical methods, including those used to control for confounding.

Answer:Categorical variables were analyzed using the Pearson Chi-square test or Fisher’s exact test. Continuous variables were analyzed using t-tests or Mann-Whitney U tests. Crucially, no multivariable logistic regression or propensity score matching was performed to adjust for confounding. This decision was made because the limited sample size (n=67) and the small number of specific outcome events would lead to statistical overfitting and unstable models. Results are presented as unadjusted associations.

(b) Describe any methods used to examine subgroups and interactions.

Answer: Subgroup analyses were not performed due to sample size constraints.

(c) Explain how missing data were addressed.

Answer: Patients with incomplete operative or baseline radiological records were excluded prior to analysis. There were no substantial missing data for the primary variables of interest in the final cohort.

(d) If applicable, explain how loss to follow-up was addressed.

Answer: Patients lost to follow-up within the first postoperative month were excluded from the study.

(e) Describe any sensitivity analyses.

Answer: None performed due to the descriptive nature of the unadjusted comparisons.

**Results**

13. Participants: (a) Report numbers of individuals at each stage of study—eg, numbers potentially eligible, examined for eligibility, confirmed eligible, included in the study, completing follow-up, and analyzed. (b) Give reasons for non-participation at each stage. (c) Consider use of a flow diagram.

Answer: The final study cohort included 67 patients (33 in TCC, 34 in EEA). We did not include a flow diagram as the consecutive enrollment process was straightforward and standard for retrospective reviews.

14. Descriptive Data: (a) Give characteristics of study participants (eg, demographic, clinical, social) and information on exposures and potential confounders. (b) Indicate number of participants with missing data for each variable of interest. (c) Summarize follow-up time (eg, average and total amount).

Answer: Detailed baseline characteristics, including age, tumor volume, QST/Kassam/Puget classifications, and preoperative visual/endocrine deficits, are summarized in Table 1 and Table 2. The groups were well-balanced in most anatomical parameters. There were no missing primary outcome data. The median follow-up time for the entire cohort is reported in the Results/Table 3 (TCC: median 35 months; EEA: median 31.5 months).

15. Outcome Data: Report numbers of outcome events or summary measures.

Answer: The EEA group had significantly higher radiographic GTR rates (82.4% vs. 45.5%, p<0.01) and better 6-month visual field MD improvement (62.5% vs. 28.0%, p=0.016). Furthermore, the EEA group experienced a lower rate of new-onset panhypopituitarism at 6 months (14.7% vs 39.4%, p=0.022) and lower incidences of 30-day transient diabetes insipidus and electrolyte imbalances (all p<0.05). Postoperative CSF leak occurred in 4 patients in the EEA group and 1 in the TCC group, though this difference was not statistically significant.

16. Main Results: (a) Give unadjusted estimates and, if applicable, confounder-adjusted estimates and their precision (eg, 95% confidence interval). Make clear which confounders were adjusted for and why they were included. (b) Report category boundaries when continuous variables were categorized. (c) If relevant, consider translating estimates of relative risk into absolute risk for a meaningful time period.

Answer: Only unadjusted estimates (p-values from bivariate tests) are provided. As explicitly stated in the Methods and Limitations sections, confounder-adjusted estimates (e.g., multivariable regression) were not calculated due to the limited sample size, which precluded robust multivariable modeling. Therefore, all findings represent unadjusted associations and do not imply definitive causal superiority.

17. Other Analyses: Report other analyses done—eg, analyses of subgroups and interactions, and sensitivity analyses.

Answer: No additional subgroup or sensitivity analyses were performed.

**Discussion**

18. Key Results: Summarize key results with reference to study objectives.

Answer: Endoscopic resection offers superior surgical outcomes for craniopharyngioma compared to open craniotomy, including higher rates of complete resection, greater symptom relief, faster postoperative recovery, and an improved safety profile.

19. Limitations: Discuss limitations of the study, taking into account sources of potential bias or imprecision. Discuss both direction and magnitude of any potential bias.

Answer: The study's retrospective design and single-center are limitations. Potential biases include selection bias and lack of control for confounding variables. These factors may limit the generalizability of the findings.

20. Interpretation: Give a cautious overall interpretation of results considering objectives, limitations, multiplicity of analyses, results from similar studies, and other relevant evidence.

Answer: In summary, for patients with craniopharyngioma meeting the appropriate indications, endoscopic endonasal surgery demonstrates significant advantages in improving the rate of gross total resection, promoting postoperative neurological recovery, and reducing short-term complications. The realization of these advantages depends on clear endoscopic visualization, precise anatomical identification, and reliable skull base reconstruction techniques. Future multicenter, prospective studies with long-term follow-up are needed to further elucidate the impact of different surgical strategies on patients' long-term prognosis.

21. Generalisability: Discuss the generalisability (external validity) of the study results.

Answer: For patients with craniopharyngioma, we believe that clinical decision-making must adhere to the principle of individualization, comprehensively evaluating factors including tumor growth pattern, size, and its relationship to the optic chiasm and vasculature. A predominantly midline tumor confined to the sellar region often favors an endoscopic endonasal approach (EEA), whereas lateral extension, frontal base involvement, or intraventricular growth may warrant transcranial or combined approaches. Approach selection is primarily anatomy-driven, based on tumor topography, hypothalamic involvement, and vascular relationships. We recommend that clinical teams carefully select the surgical approach based on the specific anatomical characteristics of the tumor and their own technical expertise. Further studies with larger and more diverse populations are needed to validate these findings.

**Other Information**

22. Funding: Give the source of funding and the role of the funders for the present study and, if applicable, for the original study on which the present article is based.

Answer: Medical New Technology Fund of Affiliated Hospital of Xu zhou Medical University (no: 2023-27C).

23. Ethical Approval: State the ethical approval of the study.

Answer: The study was approved by the institutional review board of the Affiliated Hospital of Xuzhou Medical University, with the approval number XYFY2025-KL421-01.
